# Knowledge, Attitudes, Practices and Beliefs about Medical Male Circumcision (MMC) among a Sample of Health Care Providers in Haiti

**DOI:** 10.1371/journal.pone.0134667

**Published:** 2015-08-03

**Authors:** Jessy G. Dévieux, Anshul Saxena, Rhonda Rosenberg, Jeffrey D. Klausner, Michèle Jean-Gilles, Purnima Madhivanan, Stéphanie Gaston, Muni Rubens, Harry Theodore, Marie-Marcelle Deschamps, Serena P. Koenig, Jean William Pape

**Affiliations:** 1 AIDS Prevention Program, Florida International University, Miami, Florida, United States of America; 2 University of California Los Angeles, Los Angeles, California, United States of America; 3 Haitian Study Group for Kaposi’s Sarcoma and Opportunistic Infections (GHESKIO), Port-au-Prince, Haiti; 4 Division of Global Health Equity, Brigham and Women’s Hospital, Boston, Massachusetts, United States of America; 5 Center for Global Health, Weill Cornell Medical College, New York, New York, United States of America; International AIDS Vaccine Initiative, UNITED STATES

## Abstract

**Background:**

Haiti has the highest number of people living with HIV infection in the Caribbean/Latin America region. Medical male circumcision (MMC) has been recommended to help prevent the spread of HIV. We sought to assess knowledge, attitudes, practices and beliefs about MMC among a sample of health care providers in Haiti.

**Methods:**

A convenience sample of 153 health care providers at the GHESKIO Centers in Haiti responded to an exploratory survey that collected information on several topics relevant to health providers about MMC. Descriptive statistics were calculated for the responses and multivariable logistic regression was conducted to determine opinions of health care providers about the best age to perform MMC on males. Bayesian network analysis and sensitivity analysis were done to identify the minimum level of change required to increase the acceptability of performing MMC at age less than 1 year.

**Results:**

The sample consisted of medical doctors (31.0%), nurses (49.0%), and other health care professionals (20.0%). Approximately 76% showed willingness to offer MMC services if they received training. Seventy-six percent believed that their male patients would accept circumcision, and 59% believed infancy was the best age for MMC. More than 90% of participants said that MMC would reduce STIs. Physicians and nurses who were willing to offer MMC if provided with adequate training were 2.5 (1.15–5.71) times as likely to choose the best age to perform MMC as less than one year. Finally, if the joint probability of choosing “the best age to perform MMC” as one year or older and having the mistaken belief that "MMC prevents HIV entirely" is reduced by 63% then the probability of finding that performing MMC at less than one year acceptable to health care providers is increased by 35%.

**Conclusion:**

Participants demonstrated high levels of knowledge and positive attitudes towards MMC. Although this study suggests that circumcision is acceptable among certain health providers in Haiti, studies with larger and more representative samples are needed to confirm this finding.

## Introduction

Among the countries in Latin America and the Caribbean, Haiti has the second highest prevalence of HIV infection and the highest number of people living with HIV [1]. According to the UNAIDS statistics, there are between 120,000 and 150,000 people living with HIV in Haiti, and the prevalence rate among adults between 15 and 49 years is 2.2%. In 2012, 7,500 people died of AIDS. Within the past few years, medical male circumcision (MMC) has been recommended by organizations such the World Health Organization (WHO) to help prevent the spread of HIV, particularly in developing countries with traditionally low rates of male circumcision and generalized heterosexual epidemics [2]. The WHO has classified Haiti among countries with an MMC prevalence of less than 20% [3]. Survey research conducted in the Dominican Republic in 2008 with a sample of male Dominicans and Haitian immigrants, found that 95% were uncircumcised [4].

MMC is the surgical removal of the penile foreskin (tissue which covers the head of the penis) performed by a trained health professional such as a doctor or nurse. The procedure can be done on infants, children, and adults. The WHO recommendation to increase MMC rates is based on scientific evidence demonstrating that MMC can reduce male acquisition of HIV. The evidence includes findings from several randomized controlled trials conducted in African countries (the key studies used samples in South Africa, Kenya, and Uganda) which showed that male acquisition of HIV infection during heterosexual intercourse was reduced by approximately 60% [5–7] by circumcision. A Cochrane review of those studies concluded that MMC reduced HIV heterosexual acquisition for men and that the evidence of efficacy and safety warranted inclusion of MMC in national HIV prevention guidelines [8].

A comparable direct protective effect for women has been difficult to demonstrate. However, in recently presented research from South Africa, HIV prevalence was found to be lower among women who reported only having a history of circumcised male partners [9]. Mathematical modeling has projected that male-to-female transmission could be reduced by 46% [10]. Recent studies have also found that MMC may have more of a protective effect than was previously understood for men who have sex with men (MSM), particularly for men who mostly practice insertive anal intercourse [11]. Additionally, evidence shows that circumcision can reduce transmission of sexually transmitted infections (STIs) such as human papillomavirus (HPV) and herpes simplex virus type-2 [12–14]. With efficacy and safety now considered well established [15–21], attention has shifted to scale-up of MMC in real-world settings and applicability to other high-prevalence regions. Although the scale-up of MMC in Africa has been substantial, it still lags behind the goals set by the Joint Strategic Action Framework of UNAIDS, PEPFAR, and the WHO. Uptake among men over age 25 has been particularly low [22]. Barriers to increasing demand for MMC in developing countries include issues such as ensuring medical safety and availability of resources, understanding and tailoring messages for different male age groups and their significant others, concerns about pain, and religious/cultural practices that may influence the acceptability and the rationale for encouraging MMC [22–26].

Few studies have explored the acceptability and knowledge of MMC among health care providers, especially in resource-limited settings. A study done among health care providers in Altagracia Province of the Dominican Republic showed that the majority of health care providers (93%) were aware of the health benefits of MMC and felt they needed more extensive training on performing the procedure[27]. However, in a study among physicians in the U.S., nearly 22% and 40% reported lack of knowledge about the risks and benefits of MMC among infants and adults, respectively [28]. Similarly, a study among 92 health care providers in the United States showed highly variable knowledge levels regarding the risks and benefits of infant MMC with only 23% correctly answering that MMC can reduce HIV acquisition by 60% [29].

A major component of the scale-up of MMC is education, including education of health care providers on the medical benefits of MMC and addressing barriers to their participation in MMC promotion [30]. The present study assessed knowledge, attitudes, practices and beliefs about.about medical male circumcision among health care providers at the GHESKIO Centers in Port-au-Prince, Haiti.

## Methods

### Participants

Between November 2011 and May 2013, 153 health care providers participating in continuing education programs at the Haitian Study Group for the Study of Kaposi Sarcoma and Opportunistic Infections (GHESKIO) Centers in Port-au-Prince, Haiti, were recruited to participate in an exploratory survey to assess knowledge, attitudes, practices and beliefs about medical male circumcision among health care providers. The purpose of the study was explained to the participants who were told that their participation was strictly voluntary. Informed consent was obtained from all participants.

### Ethics Statement

The study was approved by the GHESKIO Ethics Committee and the Florida International University (FIU) Institutional Review Board (IRB). Written informed consent was obtained from all participants.

### Measures

The data collection instrument was culturally adapted and translated from English to French from a survey developed by one of the co-authors for a similar study in Mysore, India [31]. The questionnaire was completed anonymously and took approximately 20 minutes to complete; it utilized both closed and open-ended questions and collected information about participants’ demographic characteristics, experiences with MMC (14 questions), knowledge and attitudes about MMC (11 questions), perceived advantages and disadvantages of MMC (2 questions with subquestions totaling 11 answers), opinions about who should perform MMC and the best age to perform the procedure on males (2 questions with subquestions), willingness to be trained to perform MMC (4 questions), and cost charged to perform MMC (3 questions).

### Data Analysis

Descriptive statistics were calculated from the responses, and bivariate analyses were conducted to estimate the odds ratio (OR) of the acceptability of performing MMC at age less than 1 year (infant circumcision) compared with acceptability of performing MMC at 1 year or older. A 95% confidence interval (CI) of the OR and χ^2^ analysis was used to test the statistical significance of differentials in acceptability of age to perform MMC on males.

Logistic regression analysis was conducted using the backward elimination (conditional) method to estimate the OR of age of acceptability of MMC, adjusting for other variables. In backward stepwise selection, variables are removed from the equation depending on the probability of the likelihood-ratio statistic, which is based on the conditional parameter estimates. Variables that were entered in step 1 were: gender; opinions about advantages and disadvantages of MMC (11 questions); acceptability of MMC (“Do you think that your male patients would accept circumcision for the prevention of HIV/STIs?”); and years of professional experience (dichotomized by more than or less than 5 years in the profession). For this analysis, missing values, wrong answers, and ‘don’t know’ responses were collapsed into one category. SAS software version 9.3 was used for these analyses.

Bayesian network analysis (BNA) was also conducted, followed by a sensitivity analysis (SA), to identify the minimum level of change required to increase acceptability of performing MMC at less than 1 year of age. Bayesian networks [32] are directed acyclic graphs which consists of nodes and arcs. Nodes represent random variables and each node takes up various values such as ‘true’, ‘false’, and ‘don’t know’ of a variable. Arcs represent direct probabilistic dependencies among them. This analysis is preferred when the knowledge about actual causation is incomplete and probabilities are used to describe a situation, given its various outcomes.

The BNA models described in this paper were created using the GeNIe modeling environment, developed by the Decision Systems Laboratory of the University of Pittsburgh and available at http://genie.sis.pitt.edu. Data were dichotomized by the same criteria as explained earlier and were imported into GeNIe software. The structure of the network was elaborated using a ‘Bayesian search learning algorithm,’ followed by ‘parameter learning,’ which are options in the GeNIe software. This network file was saved and imported into SamIam software (available at http://reasoning.cs.ucla.edu/samiam/index.php) for the SA.

The decision to accept performing MMC below one year of age could be influenced by the gender of the respondent, and how confident he or she felt about performing MMC after receiving training for this procedure. To examine such effects, structural equation modelling (SEM) analysis was conducted, with the hypothesis that the acceptability of performing infant circumcision at less than 1 year of age would be mediated by the role of gender on the willingness to offer MMC with adequate training, and also how one answered the question, “If you were to be asked to perform/assist in male circumcisions, would you need additional training?”

## Results

Table 1 summarizes participants’ characteristics, experiences with, and opinions about MMC. Participants were medical doctors (31%), nurses (49%), and other health care professionals (20%), including counselors, medical technologists, psychologists, and medical auxiliaries (Table 1). The mean work experience of physicians and nurses was 5.4 years (*SD* = 5.8). There were 42 male (27%) and 111 female (73%) participants. Approximately 76% of the providers responded that if MMC were promoted in Haiti, they would be willing to offer MMC services with adequate training. Approximately 76% believed that their male patients would accept circumcision for the prevention of HIV/STIs, and 59% believed that infancy was the best age to perform MMC. Respondents’ opinions about the best age to perform MMC was as follows: Infancy (0 to <1 year)- 59%; childhood (1 to 9 years)- 10%; adolescence (10 to 17 years)- 3%; adulthood (≥18 years)- 1%; all ages—16%; and missing information—11%. Fifty-nine percent believed that MMC should be offered at no cost to the patient. Approximately 31% of participants reported experience with performing and/or assisting in some capacity with the MMC procedure; out of this group, around 75% reported that they had not seen any complications or adverse events following MMC. The majority of participants (65%) believed that a general surgeon should perform MMC. After general surgeons, general practitioners (27%) and pediatricians (22%) were the preferred professionals chosen to perform MMC.

**Table 1:** Health Care Providers’ Characteristics, Experience With, and Opinions about Medical Male Circumcision.

Characteristic		N (%)	Acceptability of MMC		Bivariate OR (95% CI)	*P*-value
			age < 1 year	age ≥ 1 year		
Gender	Male	42 (27.0%)	25	16	1.0 (0.47–2.10)	0.99
	Female	111 (71.0%)	64	41	Ref.	
Duration of work or profession in years	Mean (SD)	5.36 (5.8)				
	Quartiles	2,4,7				
	> 5 years	95 (68.3%)	53	41	0.75 (0.35–1.59)	0.45
	**≤** 5 years	44 (31.7%)	26	15	Ref.	
Profession	Medical Doctor	48 (31.0%)	31	16	Ref.	
	Nurse	76 (49.0%)	39	36	0.56 (0.26–1.20)	0.13
	Counselor	8 (5.2%)	5	3	0.86 (0.18–4.10)	0.85
	Other	23 (14.8%)	14	5	1.44 (0.44–4.70)	0.54
Ever performed MMC	No	140 (91.5%)	80	55	0.91 (0.28–2.92)	0.87
	Yes	13 (8.5%)	8	5	Ref.	
Ever assisted in MMC	No	111 (72.1%)	58	45	Ref.	
	Yes	46 (29.9%)	31	15	1.60 (0.77–3.32)	0.20
	Assisting the clinician during the procedure	35				
	Patient screening	7				
	Preoperative or Postoperative preparation and care	12				
	Counselling	4				
Total number of MMC performed or assisted	0	98 (74.8%)	52	45	Ref.	0.34
	≤10	28 (21.4%)	18	10	0.64 (0.27–1.53)	
	>10	5 (3.8%)	4	1	0.29 (0.03–2.68)	
If you were to be asked to perform / assist in MMC, would you need additional training?	No	28 (19.3%)	17	11	1.05 (0.45–2.45)	0.90
	Yes	117 (80.7%)	69	47	Ref.	
What training do you think you should receive to perform/assist MMC? (multiple choice)	Theoretical	35 (24%)				
	Practical clinical training	59 (41%)				
	STI diagnosis and treatment	23 (16%)				
	Infection prevention	18 (12%)				
	Counseling	32 (22%)				
	All of the above	30 (21%)				
In your opinion, what would be the best age for male circumcision?	Infants (0 to <1 year)	89 (59%)				
	Children (1–9 years)	15 (10%)				
	Adolescents (10–16 years)	4 (3%)				
	Young men (17–24 years)	2 (1%)				
	All ages	24 (16%)				
	Missing	16 (11%)				

For most of the questions, health care providers had positive opinions and good knowledge about the advantages and disadvantages of MMC. More than 90% of participants said that MMC would improve penile hygiene, reduce STIs and would not entirely prevent HIV infection, thus reflecting accurate knowledge of MMC benefits in these aspects. Approximately 70% of participants thought that MMC would reduce HIV infection. When asked if MMC would increase or decrease sexual pleasure or reduce the risk of penile cancer, approximately 40% of participants did not know. Participants who agreed (16%) with the misconceptions that male circumcision prevents HIV infection entirely (adjusted odds ratio [AOR]: 0.18, 95% CI: 0.07–0.50), and male circumcision reduces (56%) sexual pleasure (AOR: 0.24, 95% CI: 0.07–0.83), were more likely to prefer MMC at age 1 year or older (Hosmer-Lemeshow test for goodness-of-fit: χ^2^ (1) = 0.003, *p* = 0.96; Cox-Snell *R* ^2^ = 0.10).

Table 2 shows the effects of "Willing to offer MMC," gender, and "Need for additional training" on choosing the best age to perform MMC as <1 year among the subsample of physicians and nurses (n = 124). Parameter estimates, AOR, Total, Direct and Indirect effects from the SEM analysis are shown.

**Table 2:** Effects of “Willing to offer MMC,” gender, and “Need for additional training” on choosing the best age to perform MMC as <1 year among physicians and nurses (n = 124). Parameter estimates, AOR, Total, Direct and Indirect effects from SEM^a^ analysis

Standardized Results			
**Path**	**Estimate**	**Std error**	***t*-value**
Willing to offer MMC to MMC age < 1 year	0.2	0.1	2.4**
Gender to MMC age < 1 year	0.1	0.1	0.7
Need additional training to MMC age < 1 year	-0.03	0.1	-0.3
Willing to offer MMC to Gender	-0.1	0.1	-1.1
Willing to offer MMC to Need additional training	0.1	0.1	1.5
**Standardized Effects of Willing to offer MMC**			
	**Total**	**Direct**	**Indirect**
MMC age < 1 year			
Effect	0.2	0.2	-0.01
Std error	0.1	0.1	0.01
*t*-value	2.3	2.4	-0.6
*p*-value	0.02*	0.01*	0.6
**Odds Ratio Estimates and Wald Confidence Intervals for MMC age < 1 year**			
**Effect**	**Estimate**	**95% Confidence Limits**	
Willing to offer MMC (No vs. Yes)	2.6**	1.2	5.7
Gender (Female vs. Male)	0.8	0.3	1.8
Need additional training (Yes vs. No)	0.9	0.4	2.1

*p-value < 0.05

**p-value < 0.001

^a^ Structural equation modelling

This sub-group analysis showed that participants who were willing to offer MMC if provided with adequate training were 2.5 times more likely to respond that the best age to perform MMC was less than 1 year (AOR: 2.5, 95% CI: 1.2–5.7; r_SP_ = 0.20, *p* = 0.02). Participants who responded ‘yes’ about willingness to offer male circumcision services with adequate training were nearly 2.6 times as likely to accept infant circumcision (AOR: 2.6, 95%CI: 1.15–5.71; r_SP_ = 0.20, *p* = 0.02) when compared to those who answered ‘no’. SEM analysis did not show significant mediation or moderation effects.

In BNA, the misconception that MMC prevents HIV transmission completely (#1); the perception that circumcised men are more promiscuous (#2); and the beliefs that MMC affects sexual pleasure [MMC reduces sexual pleasure (#3) or MMC increases sexual pleasure (#4)], were identified as the most significant contributors to the views about the best age for performing MMC. We conducted sensitivity analyses to determine the combination of minimum change needed to produce maximum acceptability of infant circumcision. Results showed that if the joint probability of choosing “the best age for MMC” as 1 year or older and having the mistaken belief that "MMC prevents HIV entirely" is reduced by 63% then the probability of finding MMC at less than 1 year acceptable to health care providers is increased by 35%.

## Discussion

This study makes significant contributions to the scant literature on healthcare providers’ attitudes and knowledge of MMC in Haiti. Generally, the results of this study are very encouraging. A large proportion of healthcare providers were willing to undergo training to conduct MMC in Haiti. Participants demonstrated high levels of knowledge and positive attitudes towards MMC. However, the study revealed that there is lack of adequately trained health care providers to perform MMC in Haiti.

A high percentage of participants (81%) said that they needed training in MMC and expressed willingness to receive training. Thirty-one percent reported that they had performed and/or assisted in performing MMC. This shows that, among this sample, MMC is not widely practiced in Haiti. This finding may be due to the fact that the participants (doctors and nurses) in this study were mostly primary care providers rather than surgeons who are usually the ones who perform and assist with MMC in clinical settings. The high level of willingness to perform MMC could be attributed to participants’ level of knowledge about the benefits of MMC. Around 41% of participants said that they needed practical clinical training and 24% said that they needed theoretical training.

However, studies in South Africa have shown that with a properly designed curriculum, usage of an appropriate educational strategy, and continued focus on critical areas of knowledge, health care providers can be successfully trained to perform MMC [33–35]. Training of health care providers, especially nurses, is an important component of dissemination of MMC into the population. Through task-shifting and task-sharing, trained nurses could help relieve the human resource burden in the widespread implementation of MMC in resource-limited countries such as Haiti [36]. Training should be integrated into clinical practice to promote MMC among physicians as well. Since MMC training was well accepted by the health care providers in this sample in Haiti, policy makers should consider incorporating MMC training into Haitian medical and nursing school curricula, after further assessments are conducted among a larger, more general sample.

Penile cancer affects approximately 1 in 900 uncircumcised men over their lifetime [37]. It is very well documented that if MMC is performed prior to sexual debut, it reduces the risk of developing penile and prostate cancers [37–39]; however, fewer than half the participants in the sample were aware of this fact [37–39]. This lack of knowledge about the association between MMC and reduction of penile or prostate cancer could be due to the low incidence rate of both cancers in Haiti [40–42]. Also, nearly 40% of the participants were unsure if MMC would increase or decrease sexual pleasure. Three randomized controlled trials done in Africa showed *higher* levels of sexual satisfaction after MMC [5–7]. Moreover, a detailed systematic review of the scientific literature conducted by Morris and Krieger [43] suggested that MMC has no adverse effect on sexual function, sensitivity, sexual sensation, or satisfaction. In the current study, probability analysis showed that better knowledge about the effect of MMC on sexual pleasure could have increased the acceptability of MMC even further among healthcare providers.

Participants of this study demonstrated a strong positive attitude towards MMC. For example, a majority of participants thought that there was no association between MMC and promiscuity and that MMC services should be provided free of charge. An individual’s attitudes will normally be influenced after becoming educated on the issue [44]. Thus, our finding of strong positive attitudes toward MMC may be related to high levels of general MMC knowledge.

Certain cultures believe that promoting male circumcision may be interpreted as endorsing risky behaviors [45,46]. This cultural belief is also present among many individuals in Haiti [47,48]; however, contrary to the cultural norms, participants in this study showed positive attitudes towards MMC. This finding may be due to the higher levels of overall education among the participants—all the participants were health care providers.

More than three quarters of participants thought that their patients, parents of infants and children as well as adults, would accept MMC to prevent HIV/STI. This finding is an indirect indicator of the acceptance of MMC among parents and adults in Haiti. These data approximate the results of a study done in the Dominican Republic, where 67% of adult men were willing to receive MMC and 74% of men were willing for MMC to be performed for their sons [4]. A review of studies on the acceptability of MMC in sub-Saharan Africa has also shown similar results, with 71%- 81% of parents willing to circumcise their sons [46].

Participants showed a slight preference for MMC being performed on infants under 1 year of age, though the difference was not significant. Regression analysis showed that physicians and nurses in this study preferred infant MMC as compared to other medical professionals. According to the WHO and a study by Morris et al., infant MMC is easier and safer to perform and less expensive compared to adult MMC [49,50]. Performing MMC during infancy is also ideal for clinicians as infants are less mobile and therefore, administration of local anesthesia is easier. Post-operative suturing is also not required in infant MMC, leading to faster wound healing and less complications[51]. Other advantages of infant MMC include prevention of urinary tract infections and other inflammatory foreskin conditions [50].

The age of sexual debut among males and females is low in Haiti [52], and when the circumcised adolescent male becomes sexually active, he would have significantly lower risk of acquiring HIV and other viral sexually transmitted diseases [17,50,53]. Incidence of cervical cancer in very high among Haitian females [41,42] and given the low age of sexual initiation, infant MMC would also decrease the risk of cervical cancer among adolescent female partners of circumcised males [53,54].

Results also suggest that it is possible to increase the acceptability of performing infant circumcision among health care providers if certain misconceptions are addressed. In particular, if the mistaken belief that MMC prevents HIV entirely could be even partially addressed, then an increased probability of the acceptability of performing MMC at less than 1 year of age could be expected.

Another finding of the current study was that the gender of the participants and training needs did not mediate the preferred age for performing MMC. This finding was contrary to a study by Carbery and colleagues [55] which showed that female physicians were uncomfortable in counseling and performing MMC among adult men.

Participants thought that general surgeons, pediatricians or any general practitioner should perform MMC, rather than nurses or other health care providers. However, previous studies have shown that shifting and sharing the tasks of MMC from and between physicians and non-physician providers, including nurses, could help distribute medical resources and increase MMC coverage [36,56]. This would be especially beneficial in settings with severe resource limitations such as Haiti. However, our findings indicate that significant education will be required to increase the acceptability of task-shifting and task-sharing among Haitian health care providers, and warrant study of the comparative outcomes of such a strategy implemented at the local level.

### Limitations

There were several limitations in this study. The sample size was quite small and limited to health care providers in one facility known to focus on HIV prevention. It is possible that in a more generalized setting, knowledge about MMC may not be as high and attitudes towards MMC may not be as favorable. In addition, while the study was focused on health care providers, it is possible that their opinions on the acceptability of MMC might not reflect those in the general population.

## Conclusion

Health care providers in Haiti demonstrated high levels of MMC knowledge, willingness to perform MMC, and positive attitudes towards MMC. However, the study demonstrated the lack of proper MMC training, especially among physicians and nurses. Although this study suggests that male circumcision is acceptable, and that health providers were willing to be trained to conduct MMC, more studies, with larger, more representative samples are needed to confirm this finding. Even if the health care providers, patients, and policy makers are ready for the introduction of MMC, the severely resource-limited health care system could pose a myriad of challenges to Haiti in successfully scaling-up MMC.
